# Inflammatory breast carcinoma as a model of accelerated self-metastatic expansion by intravascular growth

**DOI:** 10.1038/sj.bjc.6605251

**Published:** 2009-08-25

**Authors:** P B Vermeulen, S J Van Laere, L Y Dirix

**Affiliations:** 1 Translational Cancer Research Group Antwerp (Oncology Center Sint-Augustinus, Oosterveldlaan 24, and Pathology Laboratory, Antwerp University, Universiteitsplein 1, Wilrijk BE2610, Belgium)

**Sir,**

Enderling *et al* (2009) describe simulated tumour growth using an *in silico* model comprised of cancer stem and non-stem cells. Using this model, three kinetic parameters at the cellular level (cell death, proliferation and migration linked to the space available for growth) can be controlled to explain the population-level features of tumour growth, even in the absence of a heterogeneous environment. The results collectively show that migratory ability dominates tumour growth kinetics. Insufficient cell death or long-living non-stem progeny, both resulting in space constraints, impede migration and symmetrical stem cell division, and, in this way, tumour progression. The spatiotemporal evolution of the model is characterised by the phenomenon of self-metastasis (Norton and Massague, 2006), i.e., tumour growth with cluster formation, with only the stem-cell-initiated clusters persisting.

Inflammatory breast cancer (IBC) belongs to the clinical group of locally advanced breast cancers, with an unfavourable outcome. Although a relatively rare entity (only about 5% of all patients with breast cancer have IBC), worldwide several groups have focused their research on IBC for the following reasons: (1) the therapeutic opportunities are limited and new approaches need to be developed; (2) awareness needs to be increased among health workers because early diagnosis improves the outcome; and (3) IBC can function as a model of fast local growth, because cancer cell populations occupy large fractions of the breast gland within weeks to months, and as a model of efficient metastasis, because almost all patients have lymph node metastases and about one-third have distant metastases at diagnosis.

In this context, we would like to suggest that the biological features of IBC strongly support the *in silico* model of self-metastatic expansion as demonstrated by Enderling *et al* (2009). From a pathologist's point of view, IBC has a specific growth pattern with a central tumour mass surrounded by smaller nodules of carcinoma, which is in continuity with the central tumour and resembles the three-dimensional simulation of Figure 7 in Enderling's paper, but is also spread throughout the breast tissue and even in the dermis. The latter distant nodules are separated from the main tumour and from each other by normal breast parenchyma, and can be regarded as metastases within the breast. IBC is thus ‘less compact’ than non-IBC, leaving more available space for migration of cancer cells.

The growth pattern of IBC clearly fits the self-metastatic expansion hypothesis, and additional histological characteristics and the results of molecular studies of IBC further support this. In the breast parenchyma separating the tumour nodules, numerous intravascular tumour emboli can be observed, which is a well-established hallmark of IBC. Small areas of invasive carcinoma sometimes surround the intravascular tumour emboli. Regarding the kinetic parameters of the cellular level of the Enderling model, we propose that tumour emboli in blood vessels and lymph vessels represent significant cell movement throughout the breast gland, with a high *μ* value, through growth in the intravascular space. This intravascular growth has been observed in the MARY-X animal model of IBC and is based on strong homotypic interactions between the tumour cells by overexpression of E-cadherin (Alpaugh *et al*, 1999). Extravasation can occur actively, or, passively by volume increase resulting in rupture of the vessel wall, giving rise to a self-metastatic clone. In addition to the efficient migration of tumour cells in the vasculature of the breast gland, genome-wide gene expression profiling shows that IBC has overexpressed signalling pathways related to cell migration, which can lead to the ‘fingering morphologies’ at the edge of the main tumour mass. The insulin-like growth factor signalling is increased in IBC (Van Laere *et al*, 2007), leading to RhoC activation, one of the first molecular alterations that has been related to IBC and is responsible for increased cell motility (van Golen *et al*, 1999). NF-*κ*B up-regulation, another established molecular characteristic of IBC, enhances tumour cell migration by inducing the mesenchymal cell behaviour of epithelial cells (Van Laere *et al*, 2006).

An equally important condition in the self-metastasis model is the presence of cancer stem cells that are capable of migrating in less dense space to form separate tumour cell clusters; in the *in silico* model, only clones initiated by such stem cells survive. IBC clearly complies with these conditions as well. The lymphovascular emboli in animal models of IBC express a stem cell-like phenotype (Xiao *et al*, 2008). In human samples of IBC, the emboli are large and often have central necrosis and express CAIX as a marker of hypoxia, a condition in favour of a stem cell niche (Colpaert *et al*, 2003) The gene expression signature of IBC in patients contains stem cell gene expression patterns (Van Laere *et al*, 2008).

In conclusion, we believe that the fact that IBC has fast growth in the breast gland, combined with a specific growth pattern (intravascular tumour expansion and enhanced cell migration) and numerous metastases at diagnosis, corroborates the self-metastatic expansion model. The design of a new multimodality treatment for patients with IBC, and with breast cancer in general, probably has to implement the interactions of the treatment with the balance between proliferation and cell death, on the one hand, and migration in available space, on the other hand, taking into account both the stem and non-stem cell populations.
